# Simultaneous Sleep Stage and Sleep Disorder Detection from Multimodal Sensors Using Deep Learning

**DOI:** 10.3390/s23073468

**Published:** 2023-03-26

**Authors:** Yi-Hsuan Cheng, Margaret Lech, Richardt Howard Wilkinson

**Affiliations:** School of Engineering, RMIT University, Melbourne, VIC 3000, Australia

**Keywords:** machine learning, distributed networks, multimodal classification, multilabel classification, sleep stage detection, sleep disorder detection, decision-making networks

## Abstract

Sleep scoring involves the inspection of multimodal recordings of sleep data to detect potential sleep disorders. Given that symptoms of sleep disorders may be correlated with specific sleep stages, the diagnosis is typically supported by the simultaneous identification of a sleep stage and a sleep disorder. This paper investigates the automatic recognition of sleep stages and disorders from multimodal sensory data (EEG, ECG, and EMG). We propose a new distributed multimodal and multilabel decision-making system (MML-DMS). It comprises several interconnected classifier modules, including deep convolutional neural networks (CNNs) and shallow perceptron neural networks (NNs). Each module works with a different data modality and data label. The flow of information between the MML-DMS modules provides the final identification of the sleep stage and sleep disorder. We show that the fused multilabel and multimodal method improves the diagnostic performance compared to single-label and single-modality approaches. We tested the proposed MML-DMS on the PhysioNet CAP Sleep Database, with VGG16 CNN structures, achieving an average classification accuracy of 94.34% and $F_{1}$ score of 0.92 for sleep stage detection (six stages) and an average classification accuracy of 99.09% and $F_{1}$ score of 0.99 for sleep disorder detection (eight disorders). A comparison with related studies indicates that the proposed approach significantly improves upon the existing state-of-the-art approaches.

## 1. Introduction

Sleep is an integral part of human life. Poor sleep quality can lead to various physiological and mental health problems. Sleep experts identify two major stages of wakefulness and sleep, with sleep further subdivided into light sleep, deep sleep, and rapid eye movement (REM) behavior [1]. Good sleep quality is characterized by the deep sleep stage occupying a relatively high proportion of the sleep duration [2]. Therefore, accurate detection and analysis of sleep stages carry a heavy weight in the general assessment of a patient’s health. Traditional sleep assessment requires the patient to sleep in a testing room while wearing a set of sensors collecting physiological data of different modalities, such as electroencephalograms (EEG), electrocardiograms (ECG), and electromyographs (EMG). A typical recording time is eight hours. The physiological data are manually analyzed (scored) offline by at least two qualified assessors identifying sleep stage intervals and sleep anomalies indicating possible sleep disorders. The sleep scoring procedure follows the American Academy of Sleep Medicine [3] or the Rechtschaffen and Kales [4] standards. It is costly, time-consuming, and requires highly qualified human resources [5]. Therefore, despite their importance, sleep diagnosis centers have limited availability. A solution to this dilemma could be given by an automatic sleep scoring algorithm that can automatically analyze the multimodal recordings and identify sleep stages and sleep disorders [6].

Early sleep scoring studies have exhaustively analyzed feature-based approaches and classical classifiers such as the support vector machine (SVM), random forest (RF), or artificial neural networks (ANNs); for example, Ref. [7] reviewed sleep stage classification systems using ANNs. The performance varied depending on the recognized stages. A comparative study was presented in [8] that aimed to identify the most effective features and the most efficient algorithm to classify sleep stages. An accuracy of 98% was reported. In [9], a single EEG channel was used to identify optimal machine learning (ML) and feature extraction. Spectral linear features and an RF classifier led to the best classification performance, while ensuring real-time online processing. An extensive review of the current literature on automated sleep scoring can be found in [10,11].

Although systematic research progress towards automatic sleep classification has been observed for almost two decades, the recent advancement in machine learning technology offered a leap into new and exciting opportunities for designing highly effective sleep diagnosis algorithms. The majority of recent sleep scoring studies investigate single-label cases where the algorithm has a task to identify either the sleep stage or the sleep disorder modality. This task is predominantly conducted using single-modality data, most often EEG. There is also an emerging line of research where the scoring is derived from multiple modalities such as EEG and ECG. We refer to these methods as multimodal. Only a small number of papers challenged the simultaneous sleep stage and sleep disorder recognition task. We refer to these methods as multilabel. Limited studies have been published on the combination of multimodal- and multilabel sleep techniques.

An example of a single-modality sleep stage classification approach is given in Kim et al. [12]. The heart rate variability (HRV) signals were classified to identify three sleep stages (wake, light sleep, and deep sleep). After denoising, the fractal property feature of the HRV signals led to a 72% classification accuracy using pairwise correlation analysis. Another example is available in Fernández-Varela et al. [13], who used two EEG, one EOG, and two EMG channels to detect five sleep stages. An assembly of five CNNs, one for each modality, was used to classify the input time waveforms. Validation results based on the Sleep Heart Health Study (SHHS) [14,15] resulted in an $F_{1}$ score of 0.76. Phan et al. [16] used spectrogram features and a multitask CNN to detect the five classes of sleep stages. The Sleep EDF database [17,18] was used to detect five sleep stages. Accuracies of 82% to 83% were reported using the Sleep EDF database [17,18]. Rui et al. [19] used a multitask 2D-CNN to detect five sleep stages based on the time series features. A testing accuracy of 85% was achieved using the SHHS [14,15] and Sleep-EDF [17,18] data.

While there is a relatively large body of research on sleep stage detection, research into sleep disorder classification has resulted in a smaller number of publications. Zhuang and Ibrahim [20] developed a multi-channel Deep Learning (DL-AR) model where a set of CNNs was applied to six channels of raw signals of different modalities, including three channels of EEG (electroencephalogram) signals and one channel each of EMG (electromyogram), ECG (electrocardiogram), and EOG (electrooculogram) signals. The model was tested on the PhysioNet CAP Sleep database [18,21], yielding specificity and sensitivity scores of around 95% for eight sleep disorders. Sharma et al. [22] used wavelet-based features extracted from EOG and EMG signals to identify six sleep disorders from the PhysioNet CAP Sleep database [18,21]. The Hjorth transform parameters were classified using ensemble bagged trees, resulting in a testing accuracy of 94.3%.

### 1.1. Paper Contributions

Current multimodal sleep classification methods have a single-label character, i.e., the combined modalities are used to classify either a sleep stage or a sleep disorder. To our knowledge, our experiments are the first attempt to conduct a simultaneous multimodal- and multilabel classification of sleep data. There are no similar studies classifying sleep data on such a large scale, which includes six sleep stages, eight sleep disorders, and three data modalities (EEG, ECG, and EMG). This paper presents one of the first research studies in this area. To accomplish such a vast task, we introduce a new Multimodal and Multilabel Decision-Making System (MML-DMS) consisting of multiple interconnected classifiers identifying either the sleep stage or the sleep disorder from different sensor modalities. The information generated by these classifiers is then passed to two decision-making neural networks: one to identify the sleep stage and the other to identify the sleep disorder. The proposed method is tested by simultaneously identifying six sleep stages and eight sleep disorders from three different sensor modalities using the PhysioNet CAP Sleep database [18,21]. Despite the significant complexity of this task, the system offers a high performance that can be largely attributed to its distributed and modular character.

### 1.2. Paper Structure

Section 2 provides a detailed description of the proposed MML-DMS system for automatic sleep scoring. Section 3 describes the data and experiments used to validate the MML-DMS. The results are discussed in Section 4, and the paper is concluded in Section 5.

## 2. Materials and Methods

### 2.1. Proposed Multimodal and Multilabel Decision-Making System (MML-DMS)

The MML-DMS is a system of interconnected independent neural network classifiers or units. The connections are determined by the flow of information between the units. Each classifier conducts its own individual task and uses a different type or modality of input data. However, as a whole, the system performs the main task of simultaneous identification of a sleep stage and sleep disorder. The system modules are relatively simple in their architectures, can be independently trained in a time- and data-efficient manner, and can eventually be reused in other similar systems.

**Figure 1:**
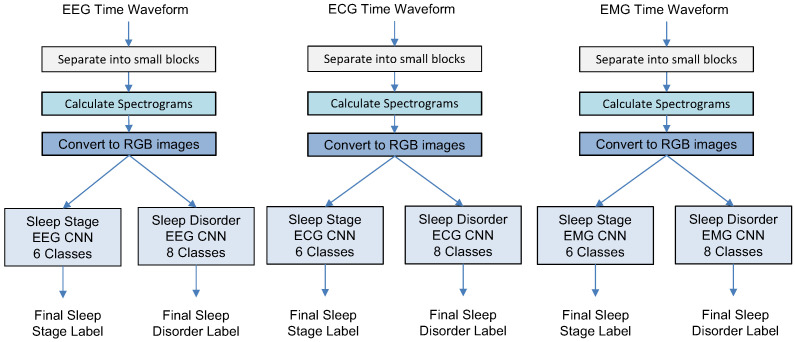
Experiment 1: Sleep stage and sleep disorder classification using a baseline approach.

**Figure 2:**
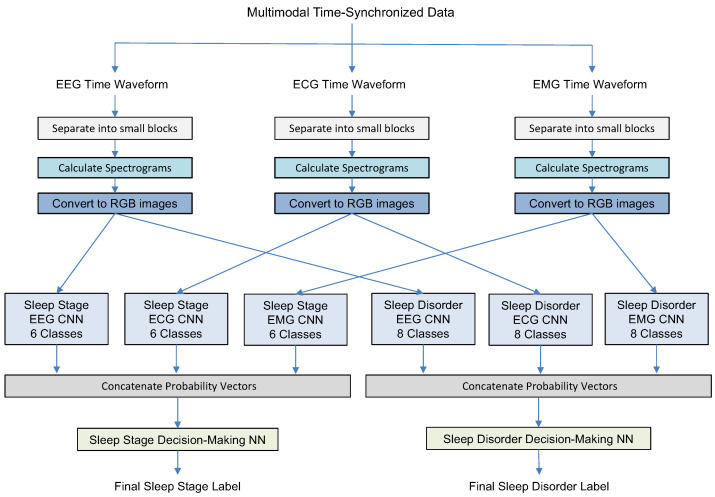
Experiment 2: Sleep stage and sleep disorder classification using MML-DMS1.

**Figure 3:**
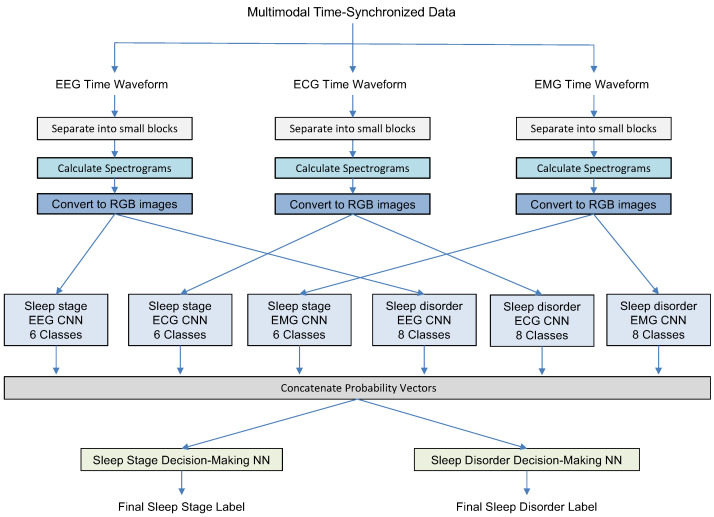
Experiment 3: Sleep stage and sleep disorder classification using MML-DMS2.

In this study, we describe three experiments designed to gradually increase the system complexity and validate the system components. All experiments have a similar first step: splitting time waveforms of different modalities into short intervals, transferring each block into a logarithmic spectrogram array, and converting it into a corresponding color RGB image. Figure 1, Figure 2 and Figure 3 illustrate how the MML-DMS concept was developed by gradually increasing its complexity and changing the interconnections between component modules. In its final form, as shown in Figure 3, the MML-DMS version, denoted as MML-DMS2, is a two-level classification procedure. At the first-level, there is an ensemble of six parallel CNN classifiers, including three networks classifying the sleep stage (one for each modality—EEG, ECG, and EMG) and three networks classifying the sleep disorder (one for each modality—EEG, ECG, and EMG).

The CNNs act as independent evaluators, directly analyzing the physiological data coming from the sensors. The probability vectors given by all six CNNs are concatenated and passed to two second-stage decision-making classifiers designed as fully connected shallow neural networks (NNs). One of the networks is trained to provide the final identification of the sleep stage and the other to identify the sleep disorder. Both stages identify the sleep stage and the sleep disorder. The difference is that in the first stage, each CNN makes decisions based on single-modality physiological data with only one label representing either the sleep stage or the sleep disorder. In contrast, the second-stage NNs use integrated sleep stage and sleep disorder information. Since the first-level CNN assessors use limited single-modality information, assessment results may vary between assessors, and their decisions may not always be correct. However, during the second stage of the classification process, the secondary NN evaluators compensate for the first-level limitations by using two-dimensional label information and arbitrating between the primary evaluators to arrive at the final sleep stage and sleep disorder labels.

### 2.2. Pre-Processing of Multimodal Data

The pre-processing steps followed were consistent across all three data modalities (EEG, ECG, and EMG). The pre-recorded time waveforms synchronized across modalities were first transformed to have the same bandwidth of 256 Hz and a sampling frequency of 512 Hz for all three modalities. The time waveforms were then divided into short-duration blocks to conduct block-by-block processing. Raw data signals sourced from the PhysioNet CAP Sleep database [18,21] represented at least eight hours of recordings labeled every 30 s with sleep stage- and sleep disorder information. However, when using 30-s non-overlapping intervals, the number of intervals was insufficient for training CNN models. Therefore, each 30-s sample was divided into overlapping 10-s intervals with a 1-s stride between subsequent blocks, resulting in a 90% overlap. The same approach was applied to all three modalities. Having such a short stride, we could generate a relatively large number of training data intervals. Since records are labeled sample-by-sample, a given interval was assumed to have the same label as the corresponding data sample. A two-dimensional spectrogram array was calculated for each interval.

### 2.3. Calculation of Amplitude Spectrograms and RGB Images

**Figure 4:**
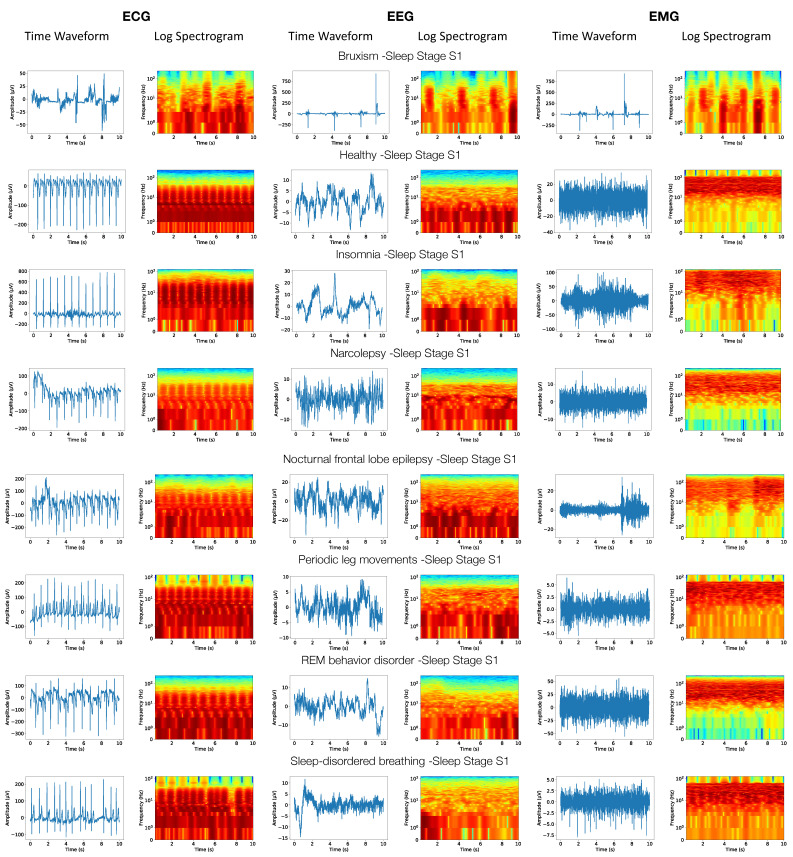
Examples of ECG-, EEG-, and EMG time waveforms and the corresponding logarithmic spectrograms for sleep stage S1 across different sleep disorders.

A two-dimensional amplitude spectrogram array was calculated for each 10 s interval using the Short-Time Fourier Transform (STFT). It was conducted the same way for all modalities to facilitate synchronized processing. By comparing the linear and the logarithmic frequency scales, it was experimentally determined that the logarithmic frequency scale led to better classification outcomes. Therefore, the spectrograms were generated using the logarithmic frequency scale, while the time scale was linear. Finally, the spectrogram arrays were converted into color RGB images using the “jet” colormap [23]. The color intensity values of the RGB images were normalized separately for each modality, with the minimum and maximum values corresponding to the average minima and maxima calculated for all images representing a given modality. Figure 4 shows examples of the original waveforms for different modalities and the corresponding RGB images representing different sleep stages and disorders. The RGB images were used to train the first-level classifiers of the proposed MML-DMS. Through visual inspection of these images, differences can be observed between the visual patterns for sleep stages and sleep disorders. These differences are difficult to comprehend by human observers. However, this study shows that CNNs can learn these differences to provide an automatic classification of sleep data.

It should be noted that the wavelet transform [24,25] is a very interesting alternative to the STFT. We used the STFT as it could be more efficiently implemented in real-time, and it is an industry-standard for real-time processing with widely available processing platforms and tools.

### 2.4. CNN Classifiers

The MML-DMS included six CNN classifiers. Each classifier was trained to recognize either a sleep stage or a sleep disorder from a single modality (EEG, ECG, or EMG). The sleep stage identification included six categories: wake (W), four sleep levels (from light sleep to deep sleep denoted S1, S2, S3, and S4, respectively), and rapid eye movement (R). At the same time, the sleep disorder identification included eight categories: normal sleep (N), Bruxism (B), insomnia (I), narcolepsy (Na), nocturnal frontal lobe epilepsy (Nf), periodic leg movements (P), REM behavior disorder (Rd) and sleep-disordered breathing (S).

The VGG16 architecture was chosen experimentally after evaluating different CNN classifiers, e.g., Inception-v3, ResNet50, and VGG16 structures, using a single classifier scenario. From the tested structures, VGG16 offered the highest classification accuracy at a reasonable computational time. In general terms, the MM-DMS is a modular classification system concept that can be implemented using different architectures for the component modules.

For all CNN models, the VGG16 CNN network structure [26,27] was used. It consisted of thirteen two-dimensional convolutional layers and three fully connected layers. The activations were rectified using a rectified linear unit (ReLu) activation function, and the learning rate was set to 0.001. All CNNs were trained from scratch; no transfer learning was applied. The VGG16 architecture was chosen experimentally after evaluating several alternative options. The VGG16 structure offered the highest accuracy at a reasonable computational time.

### 2.5. Concatenation of Probability Vectors

The final decision-making networks of the MML-DMS were trained on the soft probability vectors generated by the CNN classifiers. These vectors were concatenated and passed as inputs to the NNs. For example, given *K* data categories, *M* independent CNN classifiers, and *N* images, the probability vector generated by the *j*th CNN (j=1,…,M) for image *i* (i=1,…,N) was $P_{i,j}=\left[p_{i,j,1},\ldots ,p_{i,j,K}\right]$. Therefore, the concatenated probability vectors $C_{i}$ were given as:

(1) $$\begin{array}{cc} C_{i}= & [p_{i,1,1},\ldots ,p_{i,1,K},\\ \; & \;p_{i,2,1},\ldots ,p_{i,2,K},\\ \; & \ldots \\ \; & \;p_{i,M,1},\ldots ,p_{i,M,K}]. \end{array}$$

The concatenated probability vectors and the corresponding “ground truth” data labels were passed to the decision-making NN. It was trained to provide the final sleep stage categorization label. The probability merging process required having the same number of representative images for each modality. Since the available data contained different numbers of spectrogram images for different modalities (see Table 1), the number of training images was reduced in order to have the same number of images per modality. The NN training and testing runs were repeated three times, and the average values of the performance parameters were calculated.

### 2.6. Decision-Making Neural Network (NN)

Two shallow NNs have been trained to determine the final decision: one for the final sleep stage label; and the other for the final sleep disorder label. Both NNs consisted of an input layer containing 18 nodes, 2 hidden layers, each with 128 nodes, and an output layer with 6 nodes. The ReLu function was applied to the activations from the input and hidden layers, and the SoftMax function to the activations from the output layer. To enhance its performance, the sleep stage detection NN was trained using transfer learning from a VGG16 network pre-trained on the ECG data, as described in [23]. The sleep disorder NN on the other hand was trained from scratch, and no pre-training was applied.

### 2.7. Classical Decision-Making Methods

As shown in Figure 2 and Figure 3, when arbitrating between the outcomes of different CNN classifiers, the MML-DMS used a shallow decision-making NN. To validate the NN performance, a comparison was made by replacing the NN with other classical decision-making approaches, i.e., maximum probability, average probability, and majority voting.

When using the maximum probability method, the final label was assigned to the label indicated by the largest probability across all CNN classifiers. The majority voting approach would evaluate the categories suggested by each CNN classifier and make a decision based on the category that achieved the highest vote. When all assessors disagreed, the maximum probability criterion was used. The average probability method would average the voting provided by all CNNs for all categories and choose the category that scored the highest.

### 2.8. Performance Measures

The assessment of the MML-DMS performance was based on the classification accuracy, precision, recall, and $F_{1}$ score. Given the true positive (TP), true negative (TN), false-positive (FP), and the false-negative (FN) classification outcomes, the classification accuracy was calculated using:

(2) $$A_{\mathrm{classification}}=\frac{TP+TN}{TP+TN+FP+FN}.$$

Since the training data were unbalanced across categories, the $F_{1}$ score was estimated to indicate how well the classification accuracy was distributed across categories. It was calculated using:

(3) $$F_{1}=\frac{2\cdot Recall\cdot Precision}{Recall+Precision},$$

where the recall and precision values were defined as:

(4) $$\begin{array}{cc} Recall & =\frac{TP}{TP+FN} \end{array}$$

(5) $$\begin{array}{cc} Precision & =\frac{TP}{TP+FP} \end{array}$$

## 3. Experiments and Results

### 3.1. Data Description

The MML-DMS and the baseline approaches were tested using publicly available sleep data collected by the Sleep Disorders Center of the Ospedale Maggiore of Parma, Italy, available through the PhysioNet CAP Sleep database [18,21]. It is one of the most frequently used research databases. This choice was also motivated by the fact that the data represented recordings from multimodal sensors labeled with sleep stage as well as sleep disorder. Therefore, it provided a suitable testing bed for simultaneous multimodal- and multilabel sleep scoring. In addition, the number of available recordings was sufficient to train deep learning models. The data included synchronized waveforms representing three sensor modalities (ECG, EEG, and EMG). The total number of participants was 108. For all participants, the recordings were labeled with six sleep stages: wake (W), sleep sub stages (S1 to S4), and rapid eye movement (R). The data also included labels of normal sleep (N) from sixteen participants, and seven common sleep disorders: Bruxism (B) from two people, insomnia (I) from nine people, narcolepsy (Na) from five people, nocturnal frontal lobe epilepsy (Nf) from forty people, periodic leg movements (P) from ten people, REM behavior disorder (Rd) from twenty-two people, and sleep-disordered breathing (S) from four people.

Table 1 and Table 2 list the numbers of RGB images of spectrograms across three modalities (EEG, ECG, and EMG) for the sleep stages and sleep disorders, respectively. It can be observed that the image data were imbalanced across the sleep stage and sleep disorder categories. For the sleep stage categories, the S2 category was represented by the largest number of images, followed by the W, R, S4, S3, and S1 categories. For the sleep disorder categories, the N class was represented by the largest number of images, followed by the I, Nf, Rd, P, Na, S, and B categories.

The EEG recordings included signals collected from sixteen electrodes (P1-P16) placed on the patient’s head at different positions, as shown in [18,21]. The ECG signals were collected from two electrodes, ECG1 and ECG2, placed on the patient’s chest, as shown in [18,21]. The EMG samples included EMG measurements of the submentalis muscle and bilateral anterior tibial EMG [18,21].

### 3.2. Training, Validation and Testing Procedures

The MM-DMS modules were trained in a person-independent way. However, all participants were represented in training and testing data to achieve a fair representation of person-related diversity. For each participant and for each sleep stage, the data were split into training/validation (90%) and testing (10%) subsets. These subsets were then grouped across all subjects into the total training/validation and testing sets for the sleep stage and sleep disorder classification. The training and testing of the final trained model procedure was repeated three times, each time using different training/validation and testing subsets based on the three-fold cross-validation technique. The classification results were calculated as an average of these three repeats. The experiments were conducted using the Python programming platform with 90% of the training/validation dataset used to train the model hyperparameters and 10% of the training/validation dataset to perform validation of the training process. The hyperparameters are summarized in Table 3.

The MML-DMS is a modular system of neural networks. At the first level of classification, we have convolutional neural networks (CNNs), and at the second level, we have shallow perceptron neural networks (NNs). Each network was trained independently using standard neural network training algorithms and the same set of ground truth labels (either sleep stage or sleep disorder depending on the classification task). There was no external optimization loop with an objective function for the whole system.

For the CNNs, the objective function was the standard cross entropy loss, CE, between the ground truth probabilities p(x) and network output probabilities q(x), where *x* represents the training data vectors.

(6) $$CE=-\sum_{x}p\left(x\right)\log q\left(x\right)$$

The optimization method used was stochastic gradient descent (SGD). For the shallow NNs, the objective functions and the optimization methods were the same as for CNNs, and both levels of classification used the same ground truth labels given either by the sleep stage or sleep disorder categories of the PhysioNet CAP Sleep database. The difference was that the first-level classifiers (CNNs) were trained on physical data from sensors, whereas the second-level decision-making NNs were trained on the metadata given as probability vectors generated by the first-level CNNs.

After training each of the CNNs, the output probabilities can be saved and used to train the shallow decision-making NNs. Unlike the CNNs, which classify using only a single label—either sleep stage or sleep disorder—the NNs have the advantage of making the decision based on information provided by both sleep stage and sleep disorder labels.

### 3.3. Experimental Framework

To highlight the advantages of the MML-DMS and how it compares to baseline methods, three sleep stage and sleep disorder classification experiments were conducted. We started with a basic Experiment 1, testing the baseline CNN classifiers working with a single modality. In Experiment 2, we moved to a simplified form of the MML-DMS (denoted MML-DMS1) where there was no fusion of the sleep stage and sleep disorder information. Finally, we progressed to Experiment 3, where the sleep stage and sleep disorder information was fused at the final decision-making stage of the fully developed version of the MML-DMS (denoted MML-DMS2).

### 3.4. Experiment 1

**Figure 5:**
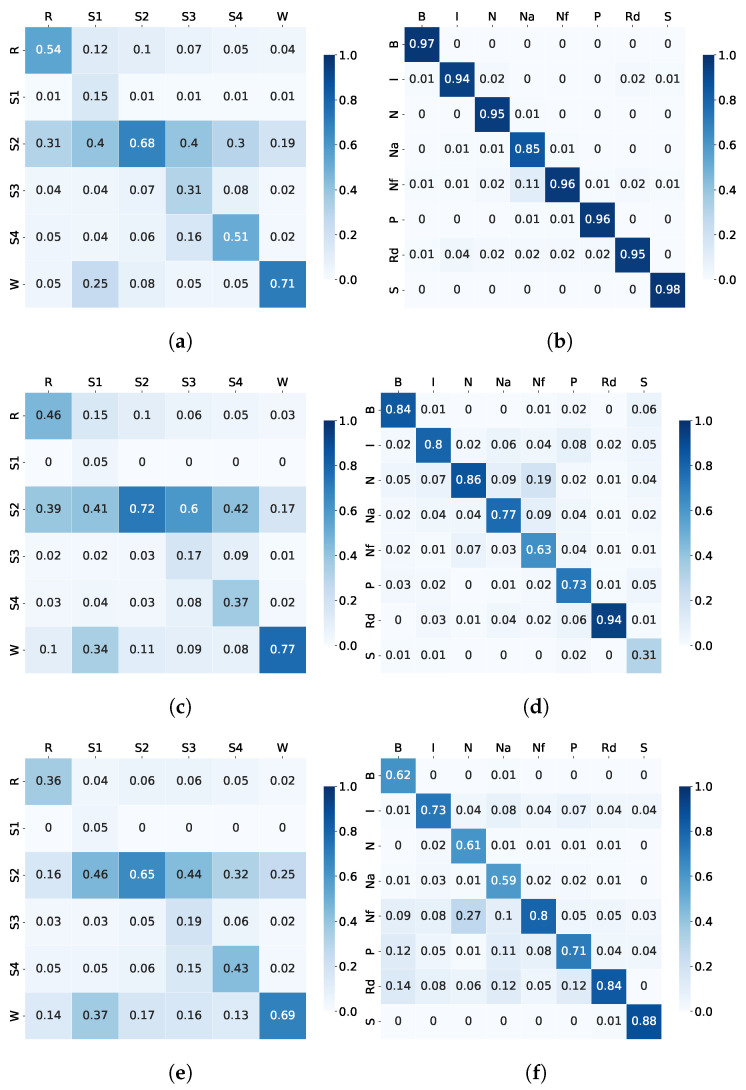
Experiment 1: Examples of confusion matrices for sleep stage and sleep disorder detection using baseline single-modality CNN classifiers: (**a**) ECG Sleep Stage detection confusion matrices; (**b**) ECG Sleep Disorder detection confusion matrices; (**c**) EEG Sleep Stage detection confusion matrices; (**d**) EEG Sleep Disorder detection confusion matrices; (**e**) EMG Sleep Stage detection confusion matrices; (**f**) EMG Sleep Disorder detection confusion matrices.

In this experiment, a simple baseline system shown in Figure 1 was created with six CNNs working in parallel to classify either sleep stage or sleep disorder based on single-modality data (EEG, ECG, or EMG). No fusion of information was applied. The resulting classification accuracy and $F_{1}$ scores are presented in Table 4, and the examples of confusion matrices are shown in Figure 5.

A comparison between sleep stage and sleep disorder detection shows that sleep disorder identification shows more than 20% higher accuracy and $F_{1}$ scores than sleep stage detection. While the sleep stage accuracy ranges between 51.4% and 57.85% and the $F_{1}$ scores from 0.4 to 0.5, for the sleep disorder, it is between 74.91% and 93.74% for the classification accuracy and between 0.74 and 0.95 for the $F_{1}$ scores. Similarly, the confusion matrices for sleep disorders show very clear diagonal patterns due to an even distribution of high accuracy across sleep disorder categories. Firstly, it could indicate that there are more distinct differences between spectral patterns of sleep disorders compared to that of sleep stages. Secondly, the data imbalances could play a less significant role in the training of disorder models than sleep stage models.

A comparison between different modalities shows that for both types of labels—sleep stage and sleep disorder—ECG signals show the highest performance, i.e., 57.85% accuracy for sleep stage and 93.74% for sleep disorder, followed by mid-performing EEG signals and finally by the lowest-performing EMG signals. It appears that ECG signals alone could be efficiently used to determine the sleep disorder. However, the sleep stage recognition scores were very low. Therefore, we needed to investigate ways of improvement to see if information fusion could be used to boost the sleep stage recognition accuracy.

### 3.5. Experiment 2

In this experiment, we test a simplified version of the MML-DMS denoted as MML-DMS1. As shown in Figure 2, it includes two levels of classification. At the first level, there are six CNN models. Three of these models are trained to identify sleep stages using only single-modality data (EEG, ECG, or EMG), and the other three to identify sleep disorders also using only single-modality data (EEG, ECG, or EMG). The probability vectors from the sleep stage classifiers are then concatenated and passed to the shallow NN (Sleep Stage Decision-making NN) trained to make the final sleep stage decision. At the same time, the probability vectors from the sleep disorder CNNs are concatenated and passed to another shallow NN (Sleep Disorder Decision-making NN) trained to decide the final sleep disorder label. The final decisions are made using a single-label approach since there is no fusion of sleep disorder information with sleep stage information.

The MML-DMS1 system allowed us to compare the multimodal information fusion with the single-modality approach used in Experiment 1. The MML-DMS1 accuracy and $F_{1}$ scores are presented in Table 5.

**Figure 6:**
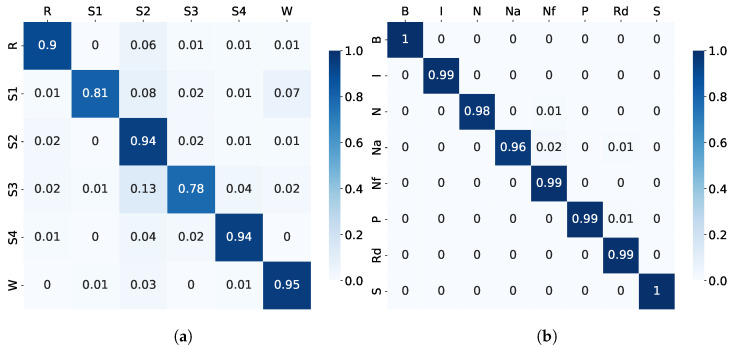
Experiment 2: Examples of confusion matrices for (**a**) sleep stage using MML-DMS1 with pre-trained NN and (**b**) sleep disorder detection using MML-DMS1 with trained-from-scratch NN.

At the same time, examples of confusion matrices are shown in Figure 6(a) and (b) for the sleep stage- and sleep disorder detection, respectively.

To determine the efficiency of NN-based decision making in comparison with other classical decision-making techniques, we have compared it with the maximum probability (MP), majority voting (MV), and average probability (AP) methods. These methods were applied within the MML-DMS structure at the second level of classification by replacing the shallow NN. The first-level CNNs remained unchanged. The results are listed in Table 5. In the case of sleep stage classification, the shallow NN trained from scratch did not perform very well, showing only 73.42% accuracy (Table 5). Therefore, a pre-trained shallow NN (PT-Shallow NN) was applied to improve the performance. In the case of sleep disorder, no pre-training of the NN was used since the trained-from-scratch NN already provided high accuracy.

It can be observed from Table 5 that the MML-DMS1 clearly outperformed the single-modality classification tested in Experiment 1. However, the sleep stage detection improvement was more significant than for the sleep disorder. The sleep stage detection achieved 91.06% accuracy, improving upon the single modalities by about 30% to 40%, whereas the sleep disorder classification achieved 98.93% accuracy, improving upon the single modalities by about 6% to 20%. A clear improvement was also observed for the $F_{1}$ scores and the confusion matrices, indicating that the multimodal approach is more robust to the data imbalances across categories. Specifically, the examples of confusion matrices for the sleep stage, as shown in Figure 6, show a much stronger diagonal pattern of high classification accuracy for individual categories than the single-modality confusion matrices shown in Figure 5.

Based on the outcomes of Experiment 2, it can be concluded that the fusion of multimodal information led to the improvement of the classification results. The classification of sleep stages was somehow more challenging and led to slightly lower results than the classification of sleep disorders. The shallow NN outperformed other classical decision-making approaches.

### 3.6. Experiment 3

In this experiment, we tested a full version of the MML-DMS denoted as MML-DMS2. It represents a multimodal as well as a multilabel approach. As shown in Figure 3, it includes two classification levels. As for the MML-DMS1, at the first level, three CNN models are trained to identify sleep, each model using only single-modality data (EEG, ECG, or EMG). Similarly, three other CNN models are trained to identify the sleep disorder from single-modality data (EEG, ECG, or EMG).

The probability vectors from all sleep stage classifiers and all sleep disorder classifiers are then concatenated and passed to the shallow NN (Sleep Stage Decision-making NN) trained to make the final sleep stage decision as well as to another shallow NN (Sleep Disorder Decision-making NN) trained to decide the final sleep disorder label. Unlike in MML-DMS1, the final decisions in MML-DMS2 are made using both multimodal and multilabel approaches, which means that in addition to fusing the multi-sensor information (EEG, ECG, and EMG), the sleep disorder information is fused with the sleep stage information.

The implementation of MML-DMS2 allowed us to compare the combined multimodal and multilabel information fusion with the single-modality approach used in Experiment 1. In addition, we could investigate the effect of adding the multilabel fusion to the multimodal approach (MML-DMS1) used in Experiment 2.

**Figure 7:**
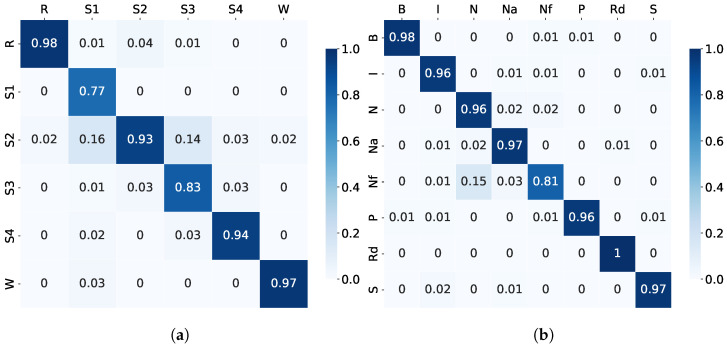
Experiment 3: Examples of confusion matrices for (**a**) sleep stage using MML-DMS2 with pre-trained NN and (**b**) sleep disorder detection using MML-DMS2 with trained-from-scratch NN.

The MML-DMS2 accuracy and $F_{1}$ scores are presented in Table 6. At the same time, the examples of the confusion matrices are shown in Figure 7(a) and (b) for the sleep stage- and sleep disorder detection, respectively. Like in Experiment 2, the shallow NN trained from scratch did not perform very well, giving only 84.89% accuracy (Table 6). Therefore, a pre-trained shallow NN (PT-Shallow NN) was applied to improve the system. No pre-training of the NN was applied for sleep disorder detection since the trained-from-scratch NN already led to high accuracy.

Table 6 shows that the MML-DMS2 achieved 94.34% accuracy for the sleep stage detection and 99.09% for the sleep disorder detection. It shows an improvement upon the MML-DMS1 of about 4% for the sleep stage and of 1% for the sleep disorder.

A clear improvement upon the MML-DMS1 was also observed for the $F_{1}$ scores and the confusion matrices, indicating that the combined multimodal and multilabel approach is even more robust to the data imbalances across categories. The examples of confusion matrices for the sleep stage, as shown in Figure 7, have very high classification accuracy for individual categories compared to the single-modality confusion matrices shown in Figure 5.

Based on the outcomes of Experiment 3, it can be concluded that the combined multimodal and multilabel information leads to an improvement in comparison with the multimodal approach and also in comparison with the single-modality baseline. The classification of sleep stages was more challenging and led to slightly lower results than the classification of sleep disorders.

## 4. Discussion

**Figure 8:**
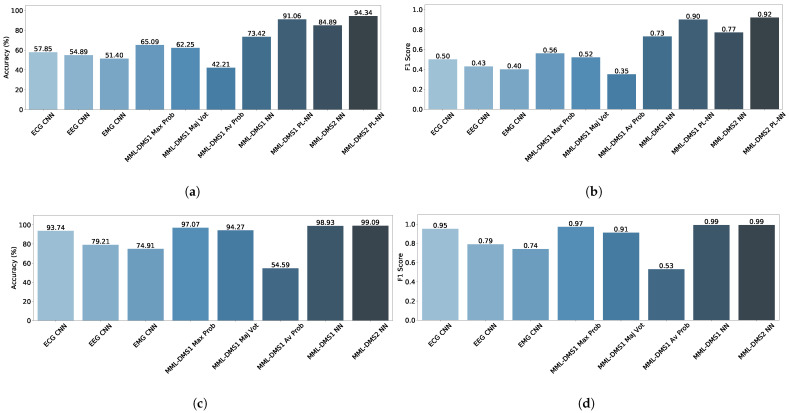
Comparison between all sleep stage and sleep disorder classification methods tested in this study: (**a**) Accuracy (%)—Sleep Stage Recognition; (**b**) $F_{1}$ Score (%)—Sleep Stage Recognition; (**c**) Accuracy (%)—Sleep Disorder Recognition; (**d**) $F_{1}$ Score (%)—Sleep Disorder Recognition.

Figure 8 shows bar graphs summarizing the outcomes of this study. In Figure 8(a) and (c), the classification accuracy is presented for the sleep stage- and sleep disorder classification, respectively, while Figure 8(b) and (d) show the corresponding $F_{1}$ scores. Each bar corresponds to a different classification approach tested in our experiments.

In Figure 8(a) and (b), pertaining to the sleep stage recognition, ten approaches are listed, including three single-modality and single-label baseline classifiers (ECG CNN, EEG CNN, and EMG CNN), five versions of the MML-DMS1 system each with a different decision-making method (MML-DMS1 MP, MML-DMS1 MV, MML-DMS1 AP, MML-DMS1 NN, and MML-DMS1 PT-NN), and two versions of the MML-DMS2—one with the trained from scratch NN (MML-DMS2 NN) and the other with the pre-trained NN (MML-DMS2 PT-NN).

In contrast, in Figure 8(c) and (d), pertaining to the sleep disorder recognition, we only have eight approaches, including three single-modality and single-label baseline classifiers (ECG CNN, EEG CNN, and EMG CNN), four versions of the MML-DMS1 system each with a different decision-making method (MML-DMS1 MP, MML-DMS1 MV, MML-DMS1 AP, and MML-DMS1 NN), and one version of the MML-DMS2 with the NN trained from scratch (MML-DMS2 NN).

The experiments demonstrated a clear advantage of combining not only the multimodal but also the multilabel information. It was confirmed by the highest performance resulting from the MML-DMS2 approach, which outperformed all other techniques and led to a 94.34% classification accuracy for the sleep stage recognition and 99.09% for the sleep disorder recognition. The $F_{1}$ scores and the confusion matrices were also consistently high, showing that the proposed modular system of networks has the capacity to compensate for the training data imbalance and give uniformly high recognition accuracy across all data categories. The second-best performance was achieved by the MML-DMS1 method offering a fusion of modalities but not the labels. It led to slightly lower classification accuracy values, i.e., 91.06% for sleep stage and 98.93% for sleep disorder classification. The highest difference was observed for the least-performing single-modality and single-label techniques. The CNN classifiers using EEG or EMG signals alone achieved around 51% to 55% accuracy for the sleep stage and about 75% to 79% for the sleep disorder recognition. Interestingly, ECG signals alone performed exceptionally well, yielding a 93.74% accuracy for the sleep disorder but only 57.85% for the sleep stage recognition. The $F_{1}$ scores and the confusion matrices corresponding to the single-modality methods were also consistently low, showing that a single CNN classifier cannot compensate for the training data imbalance.

One of the advantages of the MML-DMS is its distributed and modular character making it very versatile. The component modules are independent classifiers. Each of these classifiers uses a different combination of the input data and type of labels. The connections and data flow between modules determine the final output. It allows for either fusion or separation of specific data. Therefore, the system modules can be assembled in many different ways, and the trained units can be stored and reused depending on the task. It also means that the system can be trained with much less data, time, and lower hardware requirements compared to the large multi-branch stacked neural network structures frequently used in multimodal or multilabel problems.

One of the key factors leading to the overall high performance of the MML-DMS is the use of a shallow NN trained to arbitrate between the outcomes of an assembly of assessors (CNNs working with the single-modality data). As shown in our experiments, it outperforms other frequently used approaches, such as the maximum probability, majority voting, or average probability approaches. Each of these techniques makes certain arbitrary assumptions about how to judge the assessors. In contrast, this NN is free of such assumptions and learns directly from the data how to compensate for the potential mistakes made by the assembly of assessors.

Finally, we would like to compare the consistency of our results with other related studies. The majority of related multimodal classification methods have a single-label character, i.e., the combined modalities are used to classify either sleep stage or sleep disorder. Our experiments show one of the first attempts to conduct a simultaneous multimodal and multilabel classification of sleep data. Due to the lack of similar approaches, we present two separate tables. Table 7 shows a comparison with related sleep stage recognition studies, whereas Table 8 shows a comparison with sleep disorder classification works. We can see that for the sleep stage classification case, both of our methods outperform the best-performing study [19] by 6% (MML-DMS1) to 9% (MML-DMS2). Note that [19] classified five sleep stage categories, whereas our approach used six categories. Similarly, in sleep disorder classification, our approach outperformed the best results of [20] by 4% (MML-DMS1 and MML-DMS2).

## 5. Conclusions

In this study, we investigated the simultaneous recognition of six sleep stages and eight sleep disorder conditions from three different sensor modalities: EEG, ECG, and EMG. We proposed a new multimodal and multilabel classification system (MML-DMS). The classification outcomes derived separately for each modality by a parallel set of CNNs identifying either sleep stages or sleep disorders were fused and passed to a shallow NN to make the final decision. The system was validated using the PhysioNet CAP Sleep database and achieved 94.34% classification accuracy for sleep stage recognition and 99.09% for sleep disorder recognition.

It has to be noted that the experimental testing setup presented in this study was limited to a closed-set scenario, where the training and testing sets of samples were mutually exclusive. However, both sets represented the same groups of patients. Future research will test if the system can be generalized to accurately categorize data from patients unseen in the training process.

We demonstrated that the fusion of multimodal and multilabel information significantly improves classification outcomes compared to single-classifier and single-modality methods. Most significantly, the MML-DMS improved not only the overall classification accuracy but also the confusion matrices, leading to a uniformly high classification accuracy across all data categories. It effectively canceled out the detrimental effect of class imbalance that crippled single-modality performance. A comparison with related studies shows a significant improvement upon existing state-of-the-art techniques.

The study provided a proof of concept for simultaneous multimodal and multilabel scoring using the MML-DMS method. Due to the high complexity of the multimodal and multilabel task, MML-DMS was validated on a single database using a single type of CNN and shallow NN structure. Future research will investigate different structures of the CNN and NN classifiers and validate the proposed approach on different databases. We will also investigate improvements to sleep stage classification as it was shown to be more challenging than sleep disorder recognition.

## Figures and Tables

**Table 1:** Number of spectrogram images calculated for six sleep stages (W: wake, S1–S4: sleep sub-stages, and R: rapid eye movement), and three modalities (ECG, EEG, and EMG).

Sleep Stage	ECG	EEG	EMG
R	38002	83345	38002
S1	10405	19326	10405
S2	79338	168825	79338
S3	25229	51083	25229
S4	28179	63765	28179
W	45552	97925	45552
Total	226705	484269	226705

**Table 2:** Number of spectrogram images calculated for eight sleep disorders (N: normal sleep, B: Bruxism, I: insomnia, Na: narcolepsy, Nf: nocturnal frontal lobe epilepsy, P: periodic leg movements, Rd: REM behavior disorder, S: sleep-disordered breathing) and three modalities (ECG, EEG, and EMG).

Sleep Disorder	ECG	EEG	EMG
B	1423	25536	1423
I	18132	125116	18132
N	25599	89244	25599
Na	16764	39350	16764
Nf	58705	74328	58705
P	27330	41544	27330
Rd	67826	67575	67826
S	10926	21576	10926
Total	226705	484269	226705

**Table 3:** Hyperparameters for the VGG16 CNNs and the Shallow NNs.

Parameters	CNN	DM-Shallow NN
Optimization	SGD *	SGD *
Initial learning rate	0.001	0.001
Batch size	10	3
Maximum epochs	100	10
Early Stopping	Yes	Yes

* Stochastic Gradient Descent.

**Table 4:** Experiment 1: Classification results for the baseline single-modality CNN classifiers.

	Sleep Stage Classification		Sleep Disorder Classification	
Modality	Accuracy (%)	$F_{1}$-Score	Accuracy (%)	$F_{1}$-Score
ECG	57.85%	0.50	93.74%	0.95
EEG	54.89%	0.43	79.21%	0.79
EMG	51.40%	0.40	74.91%	0.74

**Table 5:** Experiment 2: Classification results for MML-DMS1 with different final decision-making (DM) methods (MP: maximum probability; MV: majority voting; AP: average probability; PT-Shallow NN: pre-trained NN; Shallow NN: trained-from-scratch NN).

	Sleep Stage Classification		Sleep Disorder Classification	
DM-Methods	Accuracy (%)	$F_{1}$-Score	Accuracy (%)	$F_{1}$-Score
Shallow NN	73.42%	0.73	98.93%	0.99
PT-Shallow NN	91.06%	0.90	N/A	N/A
MP	65.09%	0.56	97.07%	0.97
MV	62.25%	0.52	94.27%	0.91
AP	42.21%	0.35	54.59%	0.53

**Table 6:** Experiment 3: Classification results for MML-DMS2 using a shallow decision-making NN (PT-Shallow NN: pre-trained NN; Shallow NN: trained-from-scratch NN).

	Sleep Stage Classification		Sleep Disorder Classification	
DM-Methods	Accuracy (%)	$F_{1}$-Score	Accuracy (%)	$F_{1}$-Score
Shallow NN	84.89%	0.77	99.09%	0.99
PT-Shallow NN	94.34%	0.92	N/A	N/A

**Table 7:** A comparison with related multimodal sleep stage classification studies.

Authors	Database	Modality	Classes	Features	Method	Accuracy (%)
Kim et al. (2017) [12]	CAP	ECG, HRV	2	DFA * alpha	k-fold cross validation (k = 13)	73.6%
Fernández- Varela et al. (2018) [13]	SHHS	EEG EOG EMG	5	Time series	1D-CNN	78%
Phan et al. (2019) [16]	Sleep EDF SHHS	EEG, EOG	5	Spectrogram	Multi-task CNN	82.3%
Rui et al. (2021) [19]	Sleep EDF	EEG, EOG, EMG, ECG	5	Time series	Multi-task 2D-CNN	85%
This study	CAP	EEG, ECG, EMG	6	Log Spectrogram	MML-DMS1 MML-DMS2	91.06% 94.34%

* Detrended Fluctuation Analysis.

**Table 8:** A comparison with related multimodal sleep disorder classification studies.

Authors	Database	Modality	Classes	Features	Method	Accuracy (%)
Zhuang et al. (2022) [20]	CAP	EEG, EMG, ECG, EOG	8	Spectrogram	DL-AR	95%
Sharma et al. (2022) [22]	CAP	EOG, EMG	6	Hjorth parameters	Ensemble Bagged Trees	94.3%
This study	CAP	EEG, ECG, EMG	8	Log Spectrogram	MML-DMS1 MML-DMS2	98.93% 99.09%

## Data Availability

This study used the PhysioNet CAP Sleep database from the Sleep Disorders Center of the Ospedale Maggiore of Parma, Italy, as downloaded via physionet.org at https://physionet.org/content/capslpdb/1.0.0/ (accessed on 1 July 2020).

## Abbreviations

The following abbreviations are used in this manuscript:

AP	Average Probability
B	Bruxism
CAP	Cyclic Alternating Pattern
CNN	Convolutional Neural Network
DM	Decision-making
ECG	Electrocardiogram
EEG	Electroencephalogram
EMG	Electromyogram
EOG	Electrooculogram
FN	False Negative
FP	False Positive
HRV	Heart Rate Variability
I	Insomnia
MML-DMS	Multimodal and Multilabel Decision-making System
MP	Maximum Probability
MV	Majority Voting
N	Normal - no sleep disorder
Na	Narcolepsy
Nf	Nocturnal frontal lobe epilepsy
NN	Neural Network
P	Periodic leg movements
PT	Pre-trained
Rd	REM behavior disorder
R	Rapid eye movement
RGB	Red, Green, and Blue
S	Sleep-disordered breathing
S1-S4	Sleep stages
SGD	Stochastic gradient descent
SHHS	Sleep Heart Health Study
TL	Transfer Learning
TN	True Negative
TP	True Positive
W	Wake
